# Improvement of family-centered care in the pediatric rehabilitation ward: a participatory action research

**DOI:** 10.3389/fped.2024.1325235

**Published:** 2024-06-21

**Authors:** Taban Nematifard, Narges Arsalani, Kian Nourozi Tabrizi, Masoud Fallahi-Khoshknab, Leili Borimnejad

**Affiliations:** ^1^ Nursing and Midwifery Faculty, Kurdistan University of Medical Sciences, Sanandaj, Iran; ^2^ Iranian Research Center on Aging, Department of Nursing, University of Social Welfare and Rehabilitation Sciences, Tehran, Iran; ^3^ Nursing Department, University of Social Welfare and Rehabilitation Sciences, Tehran, Iran; ^4^ Nursing and Midwifery Care Research Center, Iran University of Medical Sciences, Tehran, Iran

**Keywords:** participatory action research, family-centered care, rehabilitation, children, hospitalization

## Abstract

**Background and aim:**

The improved life expectancy of children with disability in recent years has led to their increased request for using lifelong rehabilitation services. Family-centered care (FCC) is a model with potential positive effects on the rehabilitation of children with disability. The present study aimed at improving FCC in the pediatric rehabilitation ward.

**Methods:**

This participatory action research was conducted in 2021–2023 in the pediatric rehabilitation ward of a hospital in Tehran, Iran. Participants were 16 rehabilitation staff and 48 mothers recruited via convenient and purposive sampling methods. Data were collected using semi-structured interviews, focus group discussions, and the 20-item and the 27-item Measures of the Processes Of Care (MPOC). Data were analyzed using qualitative content analysis as well as the Kolmogorov-Smirnov and the Wilcoxon's tests.

**Findings:**

The major barrier to the implementation of FCC was staff and family limited knowledge about the importance and the benefits of FCC and the best facilitator to change was improvement of their knowledge. Therefore, an action plan based on staff and family education was designed and implemented. Participants' positive experiences of the plan were improvement of satisfaction, knowledge, collaboration, and coordination in care and their negative experiences were educational problems and dissatisfaction with the ward atmosphere. The strengths of the plan were adequate number of staff, long enough hospital stay of children, chronic course of disability, and mothers’ previous experiences. Its weaknesses were the long course of a single action plan cycle, exclusive focus on education, and the high risk of plan termination after the study. The practical problems of the study were also small physical space of the ward, transfer of some trained staff to other wards, and child discharge from the hospital.

**Conclusion:**

Staff and family limited knowledge about the importance and the benefits of FCC is a major barrier to effective FCC. Continuous education as well as family and staff collaboration may improve FCC in pediatric rehabilitation ward.

## 1 Introduction

Around 1.6 billion people, i.e., 16% of the global population, suffer from disability (1). The United Nations Development Program reported that 80% of persons with disability live in developing countries (2) and the United Nation Children's Fund reported that around 240 million of persons with disability are children (3). Disability and illness are rather distinct concepts, though they may share some certain characteristics and may be used interchangeably. Illness is the experience of a disease and is a social phenomenon that has subjective and objective aspects. The experience of an illness consists of both behavioral changes and a sense of sickness (4). Illness and disability may intersect with each other when an illness leads to a long-term disorder which significantly limits performance. Some disabilities may increase the risk of some diseases or health problems (5). Therefore, operational definitions are essential for their differentiation. According to the Convention on the Rights of Persons with Disabilities, children with disability are persons with long-term physical, mental, intellectual, or sensory damages that hinder their complete, effective, and equal participation in society (3).

Rehabilitation is an important part of disability management and a key aspect of care (6). Rehabilitation in children is a set of team-based specialized therapeutic measures and plans that aim at improving the physical, cognitive, mental, and social capabilities and skills of children and enhancing their quality of life, resilience, and participation in meaningful life activities (7).

Family-centered care (FCC) is the core of a successful rehabilitation in chronic illnesses (8). It is an innovative approach to plan, provide, and evaluate pediatric rehabilitation services which emphasizes effective collaboration with families and ensures that services are based on their needs and priorities (9). In this approach, family is an inseparable part of care and a main source of power, support, peace, and confidence in stressful conditions (10). FCC creates a familiar environment for patients (11). Family engagement in the process of rehabilitation has positive effects on treatment adherence, treatment outcomes, and prevention of re-hospitalization (12–14). The active engagement of family in care planning and provision facilitates the fulfillment of the unique needs of families and patients (10). FCC also improves functioning, satisfaction (15), and sense of control among parents (16–18), and reduces hospitalization-related fear and anxiety among children (6, 19) and families (20). It also improves emotional well-being (21, 22), physical strength (23), relationships (24–27), confidence, and joint decision making (26, 28), and reduces patients' challenges with healthcare providers (20).

Despite the necessity and the positive outcomes of family-centered rehabilitation, there are some barriers to its provision. Examples of these barriers are the dominance of paternalism in healthcare settings (15, 29, 30) and lack of standard protocols and guidelines (15, 31–34). Paternalism refers to healthcare providers' independent clinical decision making without the consent of patients or families and their belief about the appropriateness and usefulness of their decisions (15, 29, 30). Time and resource shortages also act as barriers to family-centered rehabilitation (31) and hence, advanced communication resources and systems are needed to ensure the provision of adequate attention and support to families (12, 34). Barriers to the rehabilitation of hospitalized children with disability also include organizational policies regarding FCC, managerial factors, environmental factors, factors related to the coronavirus pandemic, as well as family and staff ethical concerns, poor collaboration with each other, and limited knowledge about child rehabilitation and FCC (35).

Improvement of the quality and the outcomes of rehabilitation services for children with disability relies on the development and the use of comprehensive approaches. Of course, each child with disability has unique needs and hence, a one-size-fits-all approach would cause different challenges. Nonetheless, development of a coherent evidence-based approach can provide a good framework for the practice of healthcare providers (15, 35, 36). During literature search in online databases, we did not find any study into the improvement of the quality of FCC in the pediatric rehabilitation ward. To narrow this gap, we conducted the present study to improve FCC in the pediatric rehabilitation ward.

## 2 Methods

### 2.1 Design

This participatory action research was conducted from January 2021 to June 2023 in two coincident quantitative and qualitative phases and using the method proposed by Kemmis et al. The first author was present as a participant and facilitator in all steps of the research. The four steps of the study were planning, action, observation, and reflection (37).

**Figure 1:**
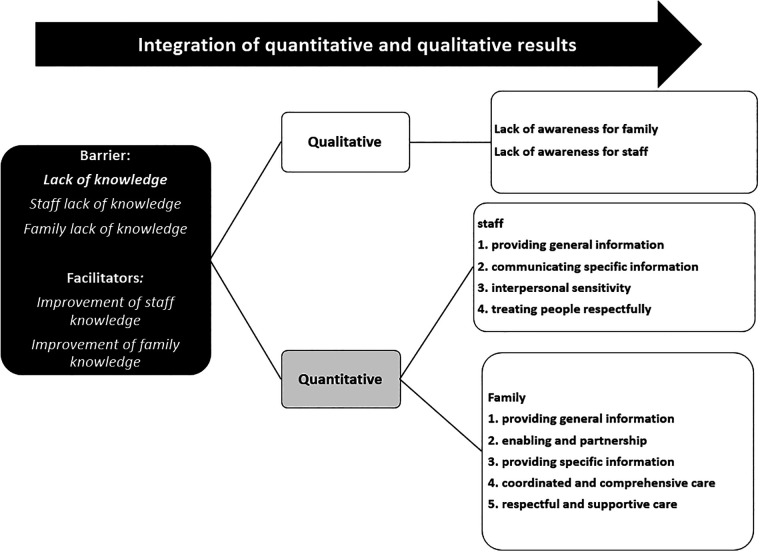
Integration of qualitative and quantitative results.

In the planning step, the barriers to FCC in the pediatric rehabilitation ward and the most appropriate strategies for their management were determined based on participants' experiences. The barriers were assessed using the quantitative questionnaire method and the qualitative interview and focus group discussion methods and the results were reported elsewhere (35). The results of quantitative and qualitative methods were combined in one session with participants through the nominal group technique (Figure 1). The nominal group technique is a good technique to combine quantitative and qualitative data in group sessions and is not affected by the problems of group dynamics associated with other group methods. In this technique, idea generation and problem solving are combined in a structured group process which encourages and enhances the participation of group members (38). During the nominal group session in the present study, nine rehabilitation staff and twelve mothers of hospitalized children collaboratively identified and discussed the most significant barriers to the implementation of FCC in the pediatric rehabilitation ward based on the results of both quantitative and qualitative methods. Subsequently, as the most important barrier was the lack of knowledge, participants proposed various strategies to improve the knowledge of rehabilitation staff and families. These strategies included the use of educational posters which highlighted FCC principles in rehabilitation as well as the use of educational pamphlets regarding the roles of rehabilitation staff and families in care. Then, the strategies were ranked and the top ranked strategies were selected for action. In the action step, an action plan, aimed at knowledge improvement, was developed and implemented. The plan was developed through group sessions with participants, where the most appropriate strategies for knowledge improvement were determined. The action plan was implemented in nine months. In the observation step, the perceived effectiveness of the nine-month action plan was assessed through interviewing participants, making field notes, and using questionnaires. In the reflection step, the strengths, weaknesses, and practical problems of the action plan and the most appropriate strategies to manage the problems and weaknesses were determined based on participants' experiences.

### 2.2 Participants and setting

The study was conducted in the pediatric rehabilitation ward of a hospital in Tehran, Iran. The hospital was the only rehabilitation hospital in Iran and families took their children there from different areas of Iran. Therefore, they experienced various problems such as loneliness, separation from family members, heavy caregiver burden, fatigue, limited perceived support, and financial strain during their child hospitalization. The ward had 13 hospitalization beds for children with the age of six months to 15 years. Based on insurance policies in Iran, each child could stay in the ward just for 63 days per year. Participants were all families who had a child with disability and a history of child hospitalization as well as all rehabilitation staff with a work experience of more than one year in the study setting. In total, 16 rehabilitation staff and 48 mothers were purposively selected in the quantitative phase and 12 mothers and nine staff were purposefully recruited with maximum variation (respecting age, gender, work experience, length of hospital stay, and child disability) in the qualitative phase (39). In the planning step of the qualitative phase, nine staff and 12 mothers of hospitalized children were involved to identify barriers and strategies for barrier management. Additionally, 16 staff and 48 mothers completed the questionnaires in the quantitative phase. In the action step, 16 staff and parents of hospitalized children participated. In the observation and the reflection steps of the qualitative phase, group interviews were conducted with 16 staff and six mothers. In the quantitative phase, 16 staff and 41 mothers stayed in the study until its end (Table 1).

**Table 1:** Participants’ characteristics.

Participants	Characteristics		*N* (%) or Mean ± SD
Staff (*n* = 16)	Average age (Years)		34.4
	Gender	Female	14 (87.5)
		Male	2 (12.5)
	Level of education	Diploma	2 (12.5)
		Bachelor’s	7 (43.75)
		Master’s	5 (31.25)
		Specialist	2 (12.5)
	Organizational position	Nurse	6 (37.5)
		Social worker	1 (6.25)
		Medical specialist	1 (6.25)
		Physical therapist	1 (6.25)
		Occupational therapist	1 (6.25)
		Speech therapist	1 (6.25)
		Nurse assistant	2 (12.5)
		Psychiatrist	1 (6.25)
		Clinical Psychologist	1 (6.25)
		Secretary	1 (6.25)
	Work experience (Years)		5.31
Mothers (*n* = 48)	Average age (Years)		35.97
	Gender	Female	48 (100)
		Male	0 (0)
	Level of education	Below diploma	2 (4.16)
		Diploma	34 (70.83)
		Bachelor’s	8 (16.66)
		Master's	4 (8.33)
	Average length of stay (Days)		49.25
	Marital status	Single	0 (0)
		Married	48 (100)

### 2.3 The quantitative phase

This phase was used in the planning and the observation steps. The Measure of the Processes Of Care (MPOC) questionnaire, developed by the CanChild Centre (40), was used to assess the process of FCC in the pediatric rehabilitation ward. MPOC-20 has 20 items in five subscales for the assessment of parents' perspectives on FCC, while the MPOC-27 has 27 items in four subscales for the assessment of staff's perspectives on FCC. The subscales of MPOC-20 are enabling and partnership (three items), providing general information (five items), providing specific information (three items), coordinated and comprehensive care for the child and family (four items), and respectful and supportive care (five items). The four subscales of the MPOC-27 are interpersonal sensitivity (thirteen items), treating people respectfully (four items), providing general information (seven items), and communicating specific information (three items) (40). These questionnaires were completed once in the planning step, i.e., before the implementation of the action plan, and once in the observation step, i.e., nine months after the initiation of the action plan. The necessary time for answering the questionnaires was 20 min. There was no information about the minimal clinically important difference for the MPOC-27 questionnaire. However, a one-point difference in the score of MPOC-20 questionnaire was determined as being clinically relevant in another study on a pediatric sample (41). For both MPOC-20 and MPOC-27 questionnaires, respondents were asked to select the best responses based on their experiences in the past year. For example, one of the questions of MPOC-20 was, “In the past year, to what extent did the people who worked with your child helped you feel competent as a parent?” In the MPOC-27 questionnaire, this question for staff was, “In the past year, to what extent did you or your organization suggested treatment/management activities that fitted with each family's need and lifestyle?” Items were scored on an eight-point scale from zero (“Not applicable”) to 7 (“To a very great extent”). Items which were scored zero were omitted and the total score was calculated through dividing the sum score of the remaining items by their number. None of the participants selected the zero response for any of the items.

The score of each subscale was calculated through summing the scores of its items and dividing the sum score by the number of its items. Therefore, the possible total score of the subscales was 0–7. Although there is no standard method for the interpretation of the scores of MPOC-20 and MPOC-27 (41), the scores of the questionnaires and their subscales can be interpreted based on the 1–7 scoring scale of their items (42).

A study reported that the intraclass correlation coefficient of the Persian MPOC-20 was 0.81 and the intraclass correlation coefficients of all its subscales were 0.60–1, which confirmed its acceptable reliability (43). The Cronbach's alpha of this instrument in the present study was 0.941. A study reported that the Persian MPOC-27 had acceptable face and content validity and found that the Cronbach's alpha values and intraclass correlation coefficients of its subscales were 0.778–0.881 and 0.75–0.83, respectively (44). We found that the Cronbach's alpha of this instrument was 0.890. The Kolmogorov-Smirnov test and the Q-Q plot showed the non-normal distribution of the data and hence, the Wilcoxon's test was used to compare the pretest and the posttest mean scores of the questionnaires. Data were analyzed via the SPSS software (v. 22.0) at a significance level of less than 0.05.

### 2.4 The qualitative phase

A qualitative study was conducted for the in-depth exploration of the barriers and strategies. Data were collected via in-depth interviews and focus group discussions. Interviews were held in a ward room and based on participants' time preferences. The main interview question for mothers and staff were, “What come into your mind when you hear ‘Care with family’ instead of ‘Care for family’?” Specific interview questions for staff were, “How do you provide care to the child in the ward?” and “What barriers do you face during your care provision?” Specific interview questions for mothers were, “How do you give care to your child in the ward?”, “How do healthcare providers provide care to your children?”, and “Which parts of care are you responsible for?” Interview questions respecting the strategies were, “What are your solutions to the mentioned problems?” (for mothers and staff) and “What resources are necessary to improve FCC in this ward?” (for staff). Based on participants' responses to these questions, probing questions like “Can you explain more?”, “Do you have any real experience in this area?”, and “Why?” were employed to collect more in-depth data. Interviews and focus group discussions lasted 30–45 and ninety minutes, respectively. The first author held all interviews with the collaboration of the corresponding author and transcribed the interviews independently. Interviews were held to explore the barriers and strategies in the planning step, while focus group discussions were held to further explore the barriers and strategies, develop the action plan, evaluate the plan, and collect data about participants' reflections.

Concurrently with data collection, data analysis was performed using Graneheim and Lundman's conventional content analysis (45). Initially, all interview and focus group discussion data were transcribed word by word and each transcript was read several times to understand its main ideas. All authors independently determined, condensed, and coded meaning units, i.e., the sentences and paragraphs that were relevant to the study aim. Then, they compared their generated codes and discussed them to reach agreement. They grouped the codes into primary subcategories and compared the subcategories and grouped them into main categories. The MAXQDA (v. 10) software was used for data management (46).

The rigor of the qualitative study was maintained using Lincoln and Guba's criteria (47). Credibility was maintained through an interview guide, member checking, and peer checking and dependability was maintained through group data analysis by all authors and documentation of all steps of the study. Confirmability was also maintained through external peer checking and providing quotations from participants’ shared experiences. In external peer checking, two experienced qualitative researchers and two PhD students in nursing evaluated and confirmed the accuracy of data analysis. Moreover, transferability was maintained by providing clear descriptions of participants' characteristics and sampling with maximum variation. Authenticity was also maintained through keeping the work as close as possible to the data and interpreting the data to create meaningful and understandable findings. The Consolidated criteria for Reporting Qualitative research were also employed to ensure the comprehensive reporting of the findings (48).

### 2.5 Ethical considerations

The Ethics Committee of the University of Social Welfare and Rehabilitation Sciences, Tehran, Iran, approved this study (code: IR.USWR.REC.1400.233). Participation was voluntary, data were kept confidential, and informed consent was obtained from all participants.

## 3 Findings

Sixty four persons participated in this study (Table 1). The findings of each step of the study are presented in what follows.

### 3.1 Findings of the planning step

The findings of this step were based on the data obtained through interviews, focus group discussions, MPOC-20, and MPOC-27. All data and their corresponding findings were checked by participants to ensure the accuracy of our interpretations. The barriers to FCC were staff lack of knowledge and family lack of knowledge and the strategies to manage these barriers were improvement of staff knowledge and improvement of family knowledge.

#### 3.1.1 Staff lack of knowledge

Staff had inadequate knowledge about the importance and the principles of FCC and provided their care services based on their personal experiences and without considering the available evidence and families' opinions. Moreover, they were reluctant to change their practice and receive the necessary education and did not value motivating interactions with families. A nurse with a work experience of five years said,

*Colleagues have inadequate knowledge about their roles and the importance of FCC and make little effort, if any, to improve their knowledge. Their FCC-related practice is based merely on their limited knowledge obtained through the Pediatric Nursing course at university. We provide FCC based on our personal experience and each staff has his/her personal approach. Some colleagues think that their practice is completely family-centered and needs no change (P. 3)*.

#### 3.1.2 Family lack of knowledge

The families of children with disability referred to the study setting from remote areas and had limited information about child care. Moreover, families, particularly those with lower educational level, did not closely adhere to staff's recommendations (due to the chronic course of their children's disability), poorly exchanged information with staff, did not report their educational needs, and reported that educations were not provided based on their needs and at an appropriate time. Families usually took care of their children at home and based on their personal methods. Although some of their personal methods were incorrect, some mothers were reluctant to learn the correct caregiving methods and resisted against staff's recommendations. However, families with higher educational level were more willing to communicate and exchange information with staff. Staff kept some families, particularly those with lower educational level, in the ward for longer periods of time in order to provide them with more education and improve their FCC-related knowledge and skills. A mother stated,

*Here, I perform routine care-related tasks for the child, [for instance] bathe him. They check and disapprove my practice by saying that I shouldn't put my child on the ground. However, I don't change my practice because I am accustomed to it (F. 1).*

Quantitative data collected using MPOC-20 and MPOC-27 also showed providing general information as the most important factor among both mothers and staff (Tables 2, 3).

**Table 2:** Comparison of the mean scores of MPOC-20 questionnaire and its subscales before and after the action plan (using the Wilcoxon test).

Time	Before	After	Mean difference (Confidence interval)	Effect size	*P* value	*α*
Subscales	Mean ± SD	Mean ± SD				
Enabling and partnership	4.06 ± 2.03	5.29 ± 1.36	1.45 (1.71–1.73)	0.76	<0.001	0.866
Providing general information	3.93 ± 1.62	5.75 ± 0.92	2.00 (1.69–2.30)	0.74	<0.001	0.785
Providing specific information about the child	4.38 ± 1.79	5.26 ± 1.21	1.34 (1.09–1.59)	0.71	<0.001	0.897
Coordinated and comprehensive care	4.67 ± 1.61	5.55 ± 1.20	1.07 (0.87–1.26)	0.75	<0.001	0.860
Respectful and supportive care	4.82 ± 1.29	5.82 ± 0.86	1.09 (0.87–1.31)	0.72	<0.001	0.767
Total	4.18 ± 1.37	5.59 ± 0.91	1.40 (1.22–1.59)	0.76	<0.001	0.946

**Table 3:** Comparison of the mean scores of MPOC-27 and its subscales before and after the intervention (using the Wilcoxon test).

Time	Before	After	Mean difference (Confidence interval)	Effect size	*P* value	*α*
Variables	Mean ± SD	Mean ± SD				
Interpersonal sensitivity	5.57 ± 0.58	6.41 ± 0.27	0.84 (0.55–1.13)	0.70	<0.001	0.83
Respectful behavior	5.81 ± 0.66	6.56 ± 0.38	0.75 (0.34–1.16)	0.53	<0.004	0.77
Provide general information	5.17 ± 0.79	6.18 ± 0.73	1.01 (0.46–1.56)	0.53	<0.004	0.71
Communication and providing specific information	5.21 ± 1.18	6.19 ± 0.83	0.98 (0.23–1.73)	0.34	<0.020	0.70
Total	5.46 ± 0.60	6.35 ± 0.35	0.89 (0.57–1.20)	0.70	<0.001	0.89

After the combination of all findings in a focus group discussion with participants, the most important barrier to the implementation of FCC was determined to be staff and family limited knowledge about the importance and the benefits of FCC and the two strategies for its management were improvement of staff knowledge and improvement of family knowledge.

#### 3.1.3 Improvement of staff knowledge

Staff were aware that each family needed unique care services; however, they had inadequate knowledge about the principles of FCC. Therefore, they highlighted the necessity of assessing educational needs, determining the necessary resources, considering the environmental culture, and providing specialized and continuous education. Moreover, they highlighted the necessity of appropriate evaluation methods to determine the level of family-centeredness of their services and improve care quality. A nurse expressed,

*Certainly, we need to implement continuous education programs in the area of child rehabilitation in order to improve staff's knowledge about FCC and their ability to provide quality FCC (P. 4).*

#### 3.1.4 Improvement of family knowledge

Participating mothers needed education but their fatigue and stress reduced their ability to effectively use the provided educational materials. Moreover, they had conflicts with ward staff due to their limited knowledge about ward routines and regulations and child care. They reported their desire to receive continuous education about ward routines and regulations, ward equipment and their use, and their roles at appropriate time and place and based on their educational level. A mother highlighted,

*They provide us with a series of education. However, I forget them and ask about them from the mothers who have children with the same problem as my child. A library in the ward would help me acquire the necessary information (F. 4).*

### 3.2 Findings of the action step

In this step, the action plan, focused on knowledge improvement, was implemented in nine months. During the planning step, we decided on holding face-to-face educational workshops for staff and providing mothers with educational booklets. Accordingly, two group workshops were held for staff using the lecture method, educational videos, and educational podcasts to improve their knowledge about FCC. Before the workshops, participants were provided with the outlines of the workshops to study them and ask their questions during the workshops. The first author held both workshops in 180 min. Participation in the workshops was not mandatory. Yet, 13 staff, out of the 16 staff in the study setting, participated in both workshops. Educational materials for those staff who had not participated in the workshops were sent through social media. The educational booklet for mothers contained materials on caregiving to children with disability and support provision to their caregivers. Twenty hard copies of the booklet were prepared and kept in the ward library. The study authors in collaboration with the ward physician, who was a pediatric neurologist, developed the booklet content using different resources (10, 24, 26, 49–55). Mothers who had a previous history of child hospitalization in the study setting and had good knowledge about child care (as determined by a nurse responsible for patient education) were identified and considered as peer mentors for other mothers. Three focus group discussions with one-month intervals were held during the action step to receive participants' feedback about the action plan. Table 4 shows the findings of this step.

**Table 4:** Results of feedback during action.

Date	Participants	Results
April 2023	All staff and families of hospitalized children (*n* = 17)	Holding a virtual workshop for staff, presenting a poster on the principles of family-centered care, providing educational pamphlets related to the principles of family-centered care
May 2023	All staff and families of hospitalized children (*n* = 12)	Revising and simplifying the text of the educational booklet, equipping the ward library with FCC-related books
June 2023	All staff and families of hospitalized children (*n* = 15)	Creating a virtual library, training the staff to use the questionnaire to assess the process of care

### 3.3 Findings of the observation step

This step was intertwined with the previous step. The overall action plan ended after nine months with data collection through questionnaires, interviews, and field notes on its effectiveness. In total, 41 mothers and 16 staff completed the questionnaires. Statistical analysis showed significant increase in the mean scores of MPOC-20 and MPOC-27 and all their subscales, particularly the providing general information subscale (*P* < 0.05) (Table 3). Qualitative assessment of participants' experiences of the action plan also resulted in the development of two main categories, namely positive experiences and negative experiences.

#### 3.3.1 Positive experiences

The positive experiences of participants respecting the action plan came into the four subcategories of improvement of knowledge, enhancement of satisfaction, improvement of collaboration in child care, and improvement of coordination in child care.

##### 3.3.1.1 Improvement of knowledge

Staff reported that the provided education helped them find answer to their questions and improved their knowledge. Knowledge improvement in turn led to evidence-based care provision and enabled staff to consider FCC and families' preferences in their practice. Role clarification during the action plan made participants aware of their responsibilities in child care and improved mothers' adherence to staff's care-related recommendations. Mothers with good care-related knowledge provided education to other mothers. Knowledge improvement and role clarification also improved care quality. A nurse stated,

*Now, I combine my knowledge and the principles of care you taught us and of course, seek families' opinions. This helps both sides have more peace and provide better care to children (P. 2).*

A mother also said,

*The educational booklet is good. I refer to it when I forget a point. Of course, my questions are answered in our sessions with other mothers (F. 3).*

##### 3.3.1.2 Enhancement of satisfaction

Participants reported that the provision of appropriate education and improvement of their knowledge had positive effects on their participation in FCC, mutual respect between staff and mothers, and information exchange between them. Improved care quality also enhanced their satisfaction and eased staff's conscience respecting care. Moreover, the action plan improved the atmosphere of the ward, boosted mothers' morale, and enhanced their overall satisfaction.

*I feel that educations have been effective. Now, I have a clean conscience after care and feel more satisfied. On the other hand, family satisfaction at the time of discharge has also enhanced and fewer problems are now reported (P. 3).*

##### 3.3.1.3 Improvement of collaboration in child care

Improvement of staff knowledge about FCC and its importance improved their supervision over the patients of their colleagues and made them help each other in medication therapy, patient and family assessment, and patient education. Provision of clear explanations to family members also fostered their collaboration and empowered them in child care.

*When I want to move my child, the nurse supervises me and quickly calls the nurse assistant. The assistant helps me move my child for example when my child wants to eliminate and we together put the child in a position to have a more comfortable elimination. I learned the principles of moving my child through studying the educational booklet and the nurse supervises and guides me (F. 4).*

##### 3.3.1.4 Improvement of coordination in child care

Education improved coordination in child care and helped staff better made the necessary appointments for child care with other hospital departments. Moreover, staff were able to appropriately manage mothers' requests for the fulfillment of children's needs. There was also more coordination among the staff of different work shifts. A staff said,

*When we call the ward to bring a child here for occupational therapy, they don't have delay as before and send the right child at the right time. Now, coordination is much better than before (P. 9).*

#### 3.3.2 Negative experiences

Participants' negative experiences of the action plan came into two main categories, namely dissatisfaction with the ward atmosphere and educational problems.

##### 3.3.2.1 Dissatisfaction with the ward atmosphere

Participants were concerned with the ethical challenges in the ward caused by the supervision of child care. Implementation of the FCC action plan and subsequent improved flexibility in some ward regulations, such as the visitation policy, caused staff dissatisfaction with family members' unrestricted attendance at the ward and caused them a sense of disorderliness in the ward. Most importantly, staff were dissatisfied with the improved knowledge of families and their resistance against accepting the responsibility of some specialized care services that were among the responsibilities of staff. A ward nurse mentioned,

*We feel someone is constantly observing our care practice. We have a sense of insecurity. Previously, the mothers did whatever we assigned to them. But now, your educations have made them resist against our responsibility delegation to them (P. 3).*

##### 3.3.2.2 Educational problems

Some staff worked night shifts and hence, could not attend the educational workshops which were held the next morning. Moreover, mothers reported that the language of the educational booklet was difficult to understand and hence, some of them needed further explanations about the educational materials. In order to manage this problem, we revised the booklet, explained its difficult terms, and used a simpler wording. Some mothers also took the booklets with them after hospital discharge and hence, the ward faced the shortage of the booklets. The heavy cost of reprinting the booklet required us to create a digital library, where participants could easily access the booklet. The transfer of the staff who had been trained during the action plan to other hospital wards and the entrance of new staff to the ward necessitated the education of the new staff, which in turn faced us with some educational problems. A ward nurse stated,

*Educations were good; but some staff could not attend educational workshops. Of course, virtual education solved this problem. There were some problems in education provision from the onset of the plan. First, the number of booklets was low. This problem was managed through establishing a digital library. Second, the language of the booklet was difficult to understand and the mothers had various questions. Thanks God, this problem was also managed (P. 3).*

### 3.4 Findings of the reflection step

The aim of this step was to determine the strengths, weaknesses, and practical problems of the action plan.

#### 3.4.1 Strengths

The main strengths of the action plan were the adequate number of staff in the study setting, staff's adequate time to allocate to children and their mothers, long enough hospital stay of children which provided a very good opportunity for providing education and receiving feedback, and mothers' previous experiences that facilitated their participation in peer group activities. The other strengths of the plan were staff's adequate time to study the educational materials before attending the workshops, provision of the educational booklet to mothers, and staff education about evidence-based practice. A nursing manager of the hospital said,

*The pediatric rehabilitation ward of our hospital had adequate number of staff and the staff had adequate time during their work shifts. This facilitated effective care provision and helped staff assess mothers' level of knowledge. Moreover, mothers with good care-related knowledge held educational sessions for other mothers. I frequently saw that more experienced mothers who had studied the booklet provided education to other mothers (P. 6).*

#### 3.4.2 Weaknesses

The long course of the action plan was one of its weaknesses. Shorter cycles with multiple rounds of evaluation and re-planning could improve the perceived effectiveness of the action plan. Moreover, the plan focused on the removal of just the most important barriers. Although the continuation of the action plan in the study setting was essential, the plan might be discontinued after the study. A nursing manager of the hospital stated,

*In my opinion, FCC needs continuity; but I'm worried about the discontinuation of this plan as soon as you finish your study. We, the humans, inherently like to perform actions more conveniently. Besides, you worked on education, while the most serious problem in the ward is the lack of physical space (P. 6).*

#### 3.4.3 Practical problems

The small physical space of the ward and the lack of a private room for providing educations and holding the interviews were the most important practical problems of the study. Moreover, as education improved care quality in the ward, the manager of the hospital transferred some trained staff of the ward to other wards. Another problem of the study was that each child could stay in the ward only for 63 days. A staff mentioned,

*The matron of the hospital and the head nurse were changed. The new head nurse was fortunately one of the staff of the ward and no significant change occurred in this process of change. But we had another problem: our patients were discharged after several weeks which caused their distance from the provided educations (P. 9).*

## 4 Discussion

The aim of this study was to improve FCC in the pediatric rehabilitation ward using evidence-based interventions. Findings showed lack of knowledge about the importance of FCC as a major barrier to successful FCC. This is in agreement with the findings of studies in Australia (8), Brazil (56), and Iran (15, 33, 57) which reported that parents and staff had limited knowledge about FCC provision in rehabilitation settings. Nonetheless, our findings revealed that some participants had limited adherence to the provided educations and resisted against change due to factors such as their overreliance on their previous experiences and routines. Similarly, families in a study felt that staff's expectations were beyond their abilities (58) and staff in another study reported significant gap between essential activities and routine FCC activities which highlighted the importance of more educations about the integration of FCC into routine clinical practice (59). Of course, some previous experiences had positive effects. For example, the chronic course of child disability and the previous experiences of child care had improved parents' coping with their problems, their ability to fulfill their needs, and their ability to provide education to their peers (60).

The reasons for participants' lack of knowledge about FCC at the beginning of the present study were education provision at inappropriate time, provision of repetitive educational materials to families, and lack of clear guidelines about FCC. Previous studies in other settings also showed that staff did not have adequate knowledge about FCC (33, 61) due to the lack of clear FCC-related guidelines (15, 62).

Based on the findings of this study, the best strategy for change was the improvement of staff and family knowledge. This is in agreement with the findings of previous studies (15, 56, 63–65). Our findings also highlighted the necessity of pre-education assessment of participants' educational needs, determination of the necessary resources and the dominant culture in the study setting, and continuous and specialized education in order to improve the perceived effectiveness of the educations and the action plan. Previous studies also reported the same finding (13, 66–68). However, our participants did not report the necessity of attention to clear guidelines. This contradicted the findings of previous studies (13, 15, 66–68). An explanation for this contradiction may be the lack of clear guidelines for FCC and lack of any FCC program in the study setting before the present study. Nonetheless, participants requested a clear method for the evaluation of the family-centeredness of the services. It seems that the influential factors on the perceived effectiveness of education largely depend on the immediate context. Using a combination of different educational methods is a good technique to improve the effectiveness of education (69, 70). We initially used the lecture method to provide FCC-related education to participants. However, previous studies reported that most components of FCC cannot be taught using the lecture method (68) and recommended the use of different teaching methods such as posters and pictures (27, 68, 71). Hence, we attempted to improve the perceived effectiveness of the study intervention using other educational methods such as booklet, posters, pamphlets, and audiovisual podcasts.

Study findings also showed that the action plan improved participants' satisfaction, knowledge, and collaboration, as well as the coordination of patient-related tasks in the study setting. Similarly, several studies reported that FCC-related education and knowledge improvement enhanced satisfaction (72, 73), improved coordination (74), and fostered collaboration among families and staff (15). Therefore, given the limited FCC-related knowledge of staff and families, education is essential to improve their knowledge and care quality (56, 73, 75, 76). On the other hand, the improvement of FCC-related knowledge in the present study was associated with some dissatisfaction. For example, improved family knowledge due to educations was associated with further role clarification and led to families' avoidance from accepting some roles and recommendations. Families in another study also reported healthcare providers' excessive expectations (58).

After the action plan, the rehabilitation staff were dissatisfied with the increased flexibility in ward regulations and subsequent disorderliness in the ward. Contrarily, a study showed that flexibility in service provision had significant positive effects on functioning and improved family participation in care (77). This contradiction may be due to the chronic course of child disability and the long hospital stay of children and their families in the rehabilitation ward. Moreover, participating staff were concerned about the sense of constant supervision of their practice. The main resources of action research also introduce this challenge as an intrusion to staff privacy and freedom (37, 78).

The action plan had some strengths and weaknesses which were mainly contextual. Unlike most inpatient pediatric settings in Iran (32, 57, 62), there was no staff shortage in the study setting and staff had adequate time to provide care and answer families' questions. Moreover, the chronic course of child disability and the long hospital stay of children provided a good opportunity for family education. However, staff transfer to other settings by the hospital manager for improving the conditions of those settings as well as the discharge of children and families during the study caused problems in the implementation of the action plan. The small physical space of the study setting for family education was another problem. This is in agreement with the findings of some previous studies (11, 79, 80). Education in the present study improved the knowledge of staff and families about the principles of FCC and their collaboration with each other in the process of care. The greater participation of staff and families in the process of care, their greater sense of competence, and their more effective information exchange improved their support for each other.

## 5 Limitations

This study was conducted using an action research design and in a small ward and hence, its findings may not be generalizable to other settings. Moreover, based on participants' opinions and needs, the focus of the study was solely on education. Given the policies of the study setting, most staff and all parents in the ward were female. Hence, all study participants were female. Some participants were also reluctant or unable to effectively share their experiences. We attempted to manage this problem by including them in both interviews and focus group discussions. Among the strengths of the study was the strong link between theory and practice in the action plan as well as the triangulation of the data collection methods.

## 6 Conclusion

This study concludes that improvement of FCC in pediatric rehabilitation ward is associated with various positive outcomes such as improvement of staff and family knowledge about FCC and improvement of their participation in the process of FCC. This study also shows that education through different educational methods can improve the perceived effectiveness of FCC. Continuous education, improvement of the physical space, and management of educational problems are necessary to improve the FCC-related knowledge of staff and family and the perceived effectiveness of FCC among staff, families, and children.

## Data Availability

The raw data supporting the conclusions of this article will be made available by the authors, without undue reservation.
